# Using Focus Groups to Validate a Pharmacy Vaccination Training Program

**DOI:** 10.3390/pharmacy3020039

**Published:** 2015-06-12

**Authors:** Mary Bushell, Hana Morrissey, Patrick Ball

**Affiliations:** School of Psychological and Clinical Sciences, Faculty of Engineering, Health Science and the Environment, Charles Darwin University, Casuarina NT 0909, Australia; E-Mails: hana.morrissey@cdu.edu.au (H.M.) ; patrick.ball@cdu.edu.au (P.B.)

**Keywords:** pharmacy, focus groups, multidisciplinary, inter-professional, pharmacy curriculum

## Abstract

**Introduction:** Focus group methodology is commonly used to quickly collate, integrated views from a variety of different stakeholders. This paper provides an example of how focus groups can be employed to collate expert opinion informing amendments on a newly developed training program for integration into undergraduate pharmacy curricula. **Materials and methods:** Four focus groups were conducted, across three continents, to determine the appropriateness and reliability of a developed vaccination training program with nested injection skills training. All focus groups were comprised of legitimate experts in the field of vaccination, medicine and/or pharmacy. **Results:** Themes that emerged across focus groups informed amendments giving rise to a validated version of a training program. **Discussion**: The rigorous validation of the vaccination training program offers generalizable lessons to inform the design and validation of future training programs intended for the health sector and or pharmacy curricula. Using the knowledge and experience of focus group participants fostered collaborative problem solving and validation of material and concept development. The group dynamics of a focus group allowed synthesis of feedback in an inter-professional manner. **Conclusions**: This paper provides a demonstration of how focus groups can be structured and used by health researchers to validate a newly developed training program.

## 1. Introduction

Focus groups are a qualitative methodology used to identify what people think, how people really behave, their, experiences and attitudes [1,2]. They use a method of group interviewing that draws advantage from communication and group interaction among participants in order to generate data [3].

Focus groups have long been used as a methodology in health care research to examine a range of issues including: interactions between health professionals and patients; the changing roles of health professions; and the organisation of health services [4]. The concept of using focus groups to evaluate both undergraduate and postgraduate educational material is not novel [5]. However adoption of the technique generally occurs once a training program, course or curriculum has been delivered to student learners [6]. This article articulates how focus groups were used to inform development and validate newly authored educational material to be delivered in undergraduate pharmacy curriculum.

In this context, the term focus group is not synonymous with working group or consultation group. Consultation groups are primarily used to elicit advice or exchange views on a guideline or policy. Working groups refer to a group of people working together temporarily until some goal is achieved; these are most commonly used to brainstorm or generate ideas. Neither a consultation group nor a working party requires prior ethical approval. Neither of these types of group are transcribed verbatim and analysed for themes in a systematic manner.

There appears to be paucity in the literature delineating how undergraduate training programs or courses, once developed, are evaluated and or validated prior to delivery. Published accounts of developed programs for undergraduate curricula commonly report evaluation post student completion of the training program. Undergraduate students commonly complete pre and post training assessments and/or surveys yielding quantitative data [7]. This commonly quantifies an increase in student knowledge, understanding, skill generation or attitudinal change after program completion [8,9]. While some publications describe how content was developed, it is consistently shown that training programs are developed in a silo fashion, with at best *ad hoc* consultation with one to two perceived and available subject matter experts. The process of development of a course or training program routinely lacks depth, transparency and transferability. As such there is a lack of published examples of how training programs are validated prior to initial delivery.

Undergraduate students are commonly asked to provide their perceptions of the value of a new course or training program via evaluation surveys post course completion. Qualitative data obtained via student interviews post course completion has also been used identify student perception of course delivery and content [10].

### Inter-Professional Focus Group

As pharmacists are part of a broader primary health care team and patient-centred care can be optimised if health professionals’ work as a cohesive team, the researchers saw value in conducting an inter-professional focus group. It was suggested that each focus group member could help contribute their knowledge, skills, and attitudes to augment and support the contributions of other participants and improve the value of feedback on the training program. Further as pharmacists embrace and expand professional services, incorporating services which have long been delivered by other health professions it is integral that collaboration, recognising others long term expertise, exists to help reduce role confusion and territorial disputes.

## 2. Experimental Section

### 2.1. Materials and Methods

To validate the proposed vaccination training program (VTP) with nested injection skills training, four focus groups were consulted, the first was conducted in the United States of America (where vaccination by pharmacists has been established for some years), the second and third focus groups took place in the Northern Territory, Australia (the country where the vaccination training program was to be embedded in undergraduate pharmacy curriculum) and the final focus group took place in Sri Lanka, South-East Asia (where the VTP would be delivered as a pilot informing future curricula directions). The focus groups consisted of; America (5), Sri Lanka (6), Australia Group 1 (3), Australia Group 2 (4), giving 19 persons overall. Each individual focus group discussion lasted approximately three hours. All were audio recorded and transcribed verbatim. In addition, discussion topics were printed on paper sized 297 mm × 420 mm (size A3) and participants were encouraged to contribute their thoughts both verbally and/or in written form by attaching a participant colour coded “Post It Note^TM^” with relevant comments to the suggested discussion topics. The posting of colour-coded notes fostered further discussion on topics and easily identified when participants presented contrasting opinions or were in unison on a topic. Feedback from all four focus groups was collated and manually coded and examined for themes. Where there was consensus in feedback, such as the suggestion to add a certain topic, or change a module title, the author of the training program revised the content to reflect focus group consensus (see Table 1). When focus groups voiced approval of the content or concept, the material was not changed, and the content was considered validated in its initial form. All amendments made and the final draft copy of the training program was sent electronically to all focus group members for final feedback.

**Table 1:** Summary of How the Training Program was Amended in Response to Focus Group Themes and Suggestions.

Guide Questions	USA Focus Group	Australia Focus Group 1	Australia Focus Group 2	Sri Lanka Focus Group	Suggestion Incorporated Training Program
Is it clear to the user how to use the manual?	Clear	Clear Could benefit from more pictures	Explain with diagram that the injections skills training in first year is the skills component of a larger embedded vaccination training program which they do not complete until 4th year	Clear	More images have been incorporated into the modules and PowerPoint presentations
Module introductions, do they introduce the reader to the topic?	Clear	Clear Suggest a forward Suggest a glossary e.g., pathogenic	Clear	Clear Abbreviations list	Glossary, abbreviations list & forward now included
Titles do they reflect the content of the modules?	Module entitled ‘anaphylaxis” suggested to be ‘retitled to anaphylaxis and emergency management’ All other titles appropriate	Clear	From module title- not clear where immunisation schedules are taught	Clear	Module revised and retitled “Managing vaccine-associated anaphylaxis in the pharmacy”
Skills taught in Year 1, 3 & 4	Appropriate	Must revisit skills competency if introduced in first year	Need to revisit injections skills, a strength of the program is revisiting the important skills	Good but if ever introduced in Sri Lanka a workshop format would be better. However assessment of skills should occur every year of the curriculum not just in 1, 3 and 4.	n/a
Competencies and the ways they are proposed to be assessed	Introduce informed consent in module one- three Objective Structured Clinical Examinations should incorporate more than skill assessment, must assess communication before and after injection	Peer assessment prior to demonstrator assessment good. Peer assessment teaches them “preceptorship” Established vaccinators assessing a strong point of the training program	Good	Approved the use of Objective Clinical Skills Examinations Must reassess how to manage anaphylaxis not just the skills of injections in 1st, 3rd and 4th year Peer review and validation a good idea Students should have an oral assessment where they talk about two vaccines in depth	OSCE modified in collaboration with USA academics Practical exercise for Cold chain expanded to include “managing cold chain in challenging environments”
The appropriateness of the selected skills and knowledge included in each of the modules and their suitability for student learning	Good Australian specific	Injection skill introduction gets students thinking that pharmacy comes with clinical skills from the commencement of the degree More case study stories ‘scaffolding’	Query how students are going to practice in real life when they will be upskilled before registered pharmacists	Must assess knowledge of adverse events specific to each vaccine.	Two additional case studies embedded in lectures and workshops
The depth of topics within each module	Infection control module too extensive	Infection control appropriate for first year and students will take the module with them throughout the curricula and into professional practice. Liked how it was population specific and regulation specific. Agreement that the modules should be updated as the practice of vaccination evolves.	Good	Credit hours to be assigned What must be removed from the course to make space for the new material	n/a
Skills, concepts, knowledge that might be missing	Serology How to screen for the evidence of immunity and vaccine preventable diseases. Safety devices and how to use them Nasal vaccination	Needle phobia Informed consent needs to be introduced in first not fourth year In fourth year complete a mock biohazard incident report as an exercise Via practical activity assess students’ knowledge on how to report an error vaccines—more depth Assess knowledge of vaccine side effects Cultural appropriateness e.g. aboriginal patients	Needle phobia and acknowledgement that some pharmacists may not feel comfortable vaccinating	List of abbreviations List of terminology Pharmacy students must spend time under the supervision of a credentialed vaccinator or in the emergency department Stock management of vaccines and adrenaline in the pharmacy	Serology education now addressed in greater depth, with added examples and knowledge assessment. Safety devices taught and used in Module 1, 2 and 3. Needle phobia now taught and students trained to provide information (supplemented with written information) to consumers about the phobia when required Students are now assessed on their knowledge of side effects via general knowledge test and oral counselling Students must integrate prior knowledge of cultural awareness in assessment scenarios
Comments on the order the training material is presented	Starting with hand washing and needle stick injury is a great choice Anaphylaxis module should come after the injection skills module	Anaphylaxis module should come after the injection skills module	Good	Good	Anaphylaxis module moved to be after the injection skills module
Other	You tube clips to support student learning Needle phobia, not all pharmacists will want to vaccinate	Linking opportunity to practice skills while on placement as nursing students do. Skill of injection itself is not hard Cold chain management a difficult concept	The skill of injection is not hard Pharmacists shouldn’t be vaccinating, anaphylaxis is a concern	Should link with further training in real time with signatures from supervisors for completing a certain number of injections Would prefer ‘intensive delivery’ not delivered over a term. The intensive should be compulsory. Certificates of competency should not be given in Sri Lanka until approved by government Vaccination training should be approved by the Health Ministry prior to inclusion in curricula outside of pilot. Should be multidisciplinary workshops with medical students invited	Four multimedia clips were developed (by a multimedia team) to assist student learning of the core skills of administering injections

### 2.2. Participant Selection

The aim of conducting focus groups for validation was to ensure that the content of the training program included all current information on vaccination, that content was sufficient for student learning and comprehension of the concepts of both injections and vaccinations and allowed for skill retention and demonstration of competency. Also that it would allow students to achieve the competency standards identified by the overarching key or professional representative organisations, such as the Australian Council of Pharmacy and the Pharmaceutical Society of Australia [11,12]. Further relevant competency standards for established vaccinator health professionals could also be met [13]. Thus the aim and objectives influenced participant selection criteria. Given that there is limited experience of pharmacists administering vaccinations [14] and subsequently developing or delivering vaccination training in Australia, the initial focus group in November 2014, for training program validation was held in The United States of America (USA), where pharmacist administered vaccinations is an established concept [15]. In the USA, all focus group participants had experience in either delivering or recently completing a vaccination training program (See Table 2). Focus group participants included two pharmacy academics both of whom deliver vaccination training to pharmacy students, two credentialed pharmacist vaccinators one responsible for up skilling pharmacists completing adjunct vaccination training, the other a pharmacy owner-operator responsible for integrating and delivering the professional service within their pharmacy. One participant in the focus group was a Doctor of Pharmacy student (Pharm.D.) who had recently completed vaccination training embedded in pharmacy curricula and had administered vaccinations while on placement.

**Table 2:** Composition of the Focus Groups.

**America**	
Pharmacy academic and pharmacist	I
Pharmacy academic and pharmacist	II
Pharmacist vaccinator and educator	III
Pharmacy owner/operator who offers vaccination service	IV
Pharm D student (completed vaccination training)	V
**Australia 1**	
Community Pharmacist	1
Nurse vaccinator	2
Aboriginal health professional /Clinical psychologist	3
Medical Doctor—no show	n/a
**Australia 2**	
Clinical Pharmacist	A
Department of Health Pharmacist	B
Community Pharmacist	C
Medical Doctor	D
Aboriginal health professional	E
**Sri Lanka**	
manager of a large pharmaceutical corporation (pharmacist)	i
a consultant physician (hospital practice)	ii
senior medical academics	iii
senior medical academics and general practitioner	iv
general practitioner	v
senior pharmacist academic	vi

The two Australian focus groups were made up of participants selected based on their current scope of clinical practice and expertise (See Table 2). Illustrative of established vaccinators in the Australian Health Care System, the focus groups had representation from the nursing, medical and Aboriginal health professionals. To help validate the training program for the Australian pharmacy setting, four registered practicing pharmacists (one clinical, two community and one employed with department of health) participated in the focus group. The inter-professional design of the focus groups allowed consultation, collaborative feedback from all current health professions involved in vaccine administration and patient care.

In Sri Lanka, focus group participants represented the medical, pharmacy and pharmaceutical industry and included the manager of a large pharmaceutical corporation (pharmacist), a consultant physician (hospital practice), two senior medical academics (one a general practitioner), one practicing general practitioner and a senior pharmacist academic. Each focus group was conducted approximately one month apart.

Participants were purposively selected to ensure representation of important elements of the research question. American and Sri Lankan participants were identified using a primary contact person who resided in each country. The contact person in both countries, via their connections was able to select participants who met inclusion criteria and who could best and most broadly inform the research question(s). All focus groups were comprised of both male and female participants and gender representation reflected the current gender trends within represented professions as such, for nursing, and pharmacy more females participated then males, while individuals representing the profession of medicine were mostly male.

A semi-structured discussion guide was developed and pretested in a pilot focus group with experts who had previously delivered post graduate vaccination training in Australia.

The focus group moderator was the researcher who had authored and developed the vaccination training program. The moderator assisted in directing and moving the discussion along and drawing out reticent responders. At the end of each discussion, the moderator verified the data collected by summarizing themes and highlighting themes that had been posted on the A3 pages. The moderator asked participants if they felt any key points had been missed. Each focus group session was electronically recorded and transcribed verbatim.

### 2.3. Ethical Considerations

The study was conducted after approval was been obtained from Charles Darwin University Human Research Ethics Committee (HREC) H14067 and Faculty of Medicine University of Ruhuna Ethical Review Committee (3.13). All focus group participants were informed of the study design and the aims of the project via a written plain language statement and verbal explanation. Written informed consent was received from all participants prior to focus group commencement. It was reiterated at the commencement of each focus group that participants were free to leave at any point if they did not wish to continue to be a part of the study.

## 3. Results

### 3.1. Support for Blended Modes of Delivery

One theme across the focus groups was the requirement of blended modes of delivery to optimise student learning. In general, participants liked the written modules; however they wanted to see more pictures to illustrate concepts.

*“The modules could benefit from more pictures, especially of the skills*”

Aus. Pharmacist A

Participants also felt the incorporation of videos and using online multiple choice questionnaires enabling automatic feedback for answers would improve student outcomes and knowledge retention.

*“There should be ‘You Tube^®^’ clips to support student learning”*

USA Pharmacist II. All other USA focus group participants agreed with this statement.

### 3.2. Omission of Material to Be Delivered

One theme consistent across both of the Australian focus groups raised by pharmacist participants was the omission or acknowledgement of needle phobia. Two pharmacists did not personally want to administer vaccinations, and strongly advocated that not every pharmacist should have to perform the clinical skill or participate in training to administer injections. There was concern that students may feel the same way as they entered a degree not anticipating direct physical patient care.

“I hate needles, wouldn’t hold a needle wouldn’t inject a needle…I personally find it scary, daunting and it does make me nervous - and if I had an option I wouldn’t”

Australian Pharmacist C

“Students shouldn’t be forced”

Australian Pharmacist A

“There needs to be education on needle phobia, it is a real fear”

Australian Aboriginal health professional 3. Australian Pharmacist 1 interjects

“yes that’s something that should be covered, students may not be expecting to vaccinate as pharmacists aren’t seen as vaccinators”

At the time of development, the module titled “Epidemiology and Vaccine Preventable Diseases” only covered in detail the vaccine preventable diseases that Australian pharmacists could currently administer vaccinations for. All four focus groups suggested the module would be improved by providing material, education and assessment on all vaccine preventable diseases or at least those identified on the current Australian immunisation schedule.

“You need to cover all vaccine preventable diseases in the training program; it is not good enough to cover only the select few that you may administer vaccines for currently”

Australian Pharmacist A

‘Even if some diseases are covered in other units you need to go over the content in a vaccination training program”

Australian Pharmacist I

“You should cover all diseases and orally assess students on their knowledge on a sample”

Sri Lankan Medical Academic iii

Further education participants identified should be covered in greater detail included; serology and how to screen for the evidence of immunity and vaccine preventable diseases. In addition it was suggested the written modules incorporated both a forward and a glossary.

### 3.3. Support for the Use of Spiral Curriculum

Across the four focus groups there was strong support for the use of spiral curricula to teach the clinical skills and applied knowledge.

“I like infection control first because it has applications in other areas of practice”

USA Pharmacist I

There was some concern that introducing the skills of injection in the first year of the degree was too early.

“injectable coincides with anatomical sites so to understand the importance of the placement of the vaccine, you have to understand the underlying anatomy to make those decisions, that’s why we have it in second year cause in first year they learn the underlying anatomy”

Australian Nurse 2 - Pharmacist 1 interjects

“First year is huge course content it can be just pushed to the side and you know like anything else creates stress so in the third and fourth year they can revisit it… refresh”

“I am concerned again about the retention from 1st to 4th year you need the assessment again in 3rd year, especially as I assume they are currently unable to demonstrate competency while on placement”

American Pharmacist I

“I like the fact the skills are reinforced and retested”

American Pharmacist III

Three focus groups voiced that the material in modules four through nine could not be taught earlier than fourth year, that is students had to have the underpinning foundation knowledge prior to undertaking the modules.

Participants voiced their approval for the learning outcomes at the commencement of each module. Further participants thought that the learning outcomes were aligned with assessments and would allow for the achievement of competency in the specified skills.

Further a strength that was voiced across three focus groups was the contextualisation of content in the country it was to be delivered. As the author had authored each individual module, participants approved the current and Australian specific content, and thought the modules could be used not only to teach pharmacy students, but teach pharmacists wishing to complete vaccination training and maintain their skills.

“It’s good how it is population and regulation specific”

Australian Pharmacist 1

## 4. Discussion

This study has drawn on participant expertise to help validate a vaccination training program with nested injection skills. The first emerging theme from participants was the articulated support for the blended modes of delivery embedded throughout the training program. Blended learning is not novel in pharmacy courses or higher education and it is being increasingly utilised to optimise learning outcome achievement [16]. The literature describes numerous accounts where blended learning programs have been shown to be superior to single delivery mode programs [17,18]. In addition to pedagogical techniques already embedded in the training program, participants voiced that additional educational technologies could be included or increased to enrich the vaccination training program. Participants suggested that the training program should include narrated visual presentations of key skills, such as drawing up from a vial and an ampoule. Consistent with what is scribed in the literature, participants further voiced making knowledge assessment online would allow for greater and timelier feedback enhancing student learning and outcomes [19].

The group dynamics of the focus groups allowed synthesis of feedback in an inter-professional manner. In day to day practice health care professionals work and learn together to deliver quality patient-centred health care. Pharmacists do not work in isolation but are part of a broader health care team. Further while the skill of administering injections is new for Australian pharmacists it is not at all novel for Australian doctors or appropriately credentialed nurses [14]. Inter-professional practice aligned with inter-professional education has been identified as a means of promoting broad levels of expertise. Participants collectively presented a broad level of expertise which worked well to help inform amendments and validation of the training program. The methodology helped provide inter-professional critique that highlighted strengths and weaknesses including omissions in the training program that should be addressed.

The deliberate attention of the focus group on the validation of the vaccination training program content, delivery and educational strategies, utilised participant skills and expertise without asking or drawing on participants perceptions about the broadening scope of practice for pharmacists in Australia. At the time the focus groups were conducted (late 2014 and early 2015) the change in scope of practice and changing jurisdictional regulations to enable pharmacists to legally vaccinate, was being debated and opposed by professional bodies such as The Royal College of General Practitioners via position statements and media appearances [20,21].

Focus group participants identified material that should be included when teaching Australian students vaccination training with nested injection skills. Participants across focus groups voiced there should be learning, teaching and knowledge assessment on all vaccine preventable diseases. This is consistent with vaccination training delivered to American students who complete the American Pharmacists Association’s (APhA) Pharmacy-Based Immunization Delivery [22].

The omission of education on needle phobia in the training program was not inadvertent, the concept was not thought of by the author. It was not until the focus groups and the repeated discussion on needle phobia by all Australian pharmacist participants, which the author thought it important to incorporate such education. Needle phobia affects between 3.5% and 10% of the population, invariably pharmacists may be affected by the disorder [23,24]. Historically, Australian pharmacists have provided care without physical contact. It became evident via the focus groups that pharmacists had commenced study and entered the pharmacy profession without envisaging that would have to administer injections. The unanticipated shift in the scope of practice pharmacists may not be well received by everyone in the profession. Further research is needed to quantify the incidence of needle phobia amongst pharmacy students and pharmacists.

There was widespread support for the use of spiral curriculum, that is, the considered iterative revisiting of topics in successive difficulty throughout the pharmacy degree. This was not surprising given that spiral curriculum has been successfully employed and widely applied to teach nursing, dentistry and medicine education and skills [25].

Using the knowledge and experience of focus group participants fostered collaborative problem solving and validation of material and concept development. Content was validated for the designated level and appropriateness of difficulty. Content was further validated for the alignment with learning outcomes and integration with prior teaching and learning. As a result of feedback from focus group participants a new learning module has been developed (needle phobia) and existing modules expanded to include key concepts (epidemiology of all vaccine preventable diseases taught). The authors believe the use of focus group methodology to validate education materials and programs offers a sound way of validating a program prior to delivery and can be adopted by other courses external to pharmacy.

### Limitations

One limitation of the study was the difficultly in recruiting Australian general practitioners (GPs) to participate in the focus group. This may be attributed in part to the voiced opposition peak professional medical bodies such as the Australian Medicines Association and The Royal Australian College of General Practitioners have against pharmacist administered vaccinations. However research has indicated in Australia that recruitment of GPs to participate in any research project is problematic [26]. This was not a limitation faced when recruiting Sri Lankan focus group participants; in contrast most participants were medical doctors. Ease of recruitment of medical doctors into the focus group may be a result of the primary contact person for recruiting participants was a longstanding, respected medical doctor in South East Asia.

## 5. Conclusions

This study outlines observations on a method for development and validation of a vaccination training program. The study demonstrates focus group methodology can be successfully used to validate a training program prior to delivery to students.
